# Gelatin 4% and hydroxyethyl starch (130/0.42) impair clot formation in a human ex vivo whole-blood resuscitation model

**DOI:** 10.1186/s13049-026-01690-6

**Published:** 2026-09-14

**Authors:** Marco Mannes, Doreen Tabea Spiegelburg, Thomas Datzmann, Anke Schultze, Andreas Bauer, Gerhard Achatz, Markus Huber-Lang, Christian Karl Braun

**Affiliations:** 1 Institute of Clinical and Experimental Trauma Immunology, Ulm University Medical Center, Ulm, Germany; 2 Department of Trauma Surgery and Orthopaedics, Reconstructive and Septic Surgery, Sportstraumatology, Trauma Surgery Research Group, German Armed Forces Hospital, Ulm, Germany; 3 Department of Pediatrics and Adolescent Medicine, Ulm University Medical Center, Ulm, Germany; 4 German Center for Child and Adolescent Health (DZKJ), Partner Site Ulm, Ulm, Germany

**Keywords:** Fluid resuscitation, Coagulation, Crystalloids, Colloids, Hydroxyethyl starch, Gelatin

## Abstract

**Background:**

Colloid solutions provide effective intravascular volume expansion but can impair hemostasis, which is particularly critical after trauma-associated hemorrhagic shock. The differential effects of specific colloids on plasmatic coagulation, platelet function and complement activation, especially when used alongside crystalloids, are still underexplored. We therefore examined graded hemodilution with gelatin or hydroxyethyl starch, alone and in combination with a balanced crystalloid, in an ex vivo human whole-blood model over time.

**Methods:**

We used a citrate- and heparin-free human ex vivo whole-blood model to assess the effects of volume dilution with a balanced crystalloid (Jonosteril), gelatin solution (Gelafundin 4%) and hydroxyethyl starch (HES 130/0.42, 6%; Tetraspan). Whole-blood was diluted at increasing ratios up to 4:6 (diluent:blood), incubated for 30 minutes at 37 °C and analyzed for blood gases, standard coagulation tests, platelet activation (CD62P), von Willebrand factor (vWF), thrombin–antithrombin complexes, complement activation and viscoelastic parameters by ROTEM (EXTEM, INTEM, FIBTEM).

**Results:**

At high dilution ratios, colloid-diluted samples exhibited more frequent macroscopic clotting and significantly lower pH with more pronounced base deficit than Jonosteril. Gelafundin, but not Jonosteril or Tetraspan, increased vWF levels over time, while CD62P expression, thrombin-antithrombin complexes and complement markers remained unchanged. ROTEM revealed a clearly hypocoagulable profile after dilution with Gelafundin or Tetraspan, most pronounced with HES, with prolonged clotting time and clot formation time, reduced a-angle and maximum clot firmness. When combining Jonosteril with increasing colloid fractions at fixed dilution, these changes followed a non-linear, ratio-dependent pattern.

**Conclusion:**

Balanced colloids, particularly HES 130/0.42, exert intrinsic, ratio-dependent adverse effects on clot formation beyond simple dilution, suggesting that hemostatic safety may depend not only on fluid type but also on the tolerated proportion of colloid within resuscitation strategies.

**Supplementary information:**

The online version contains supplementary material available at https://doi.org/10.1186/s13049-026-01690-6.

## Introduction

Severe tissue trauma and associated hemorrhage range among the most relevant causes of death in the younger population [1]. Blood loss and resulting hemorrhagic shock (HS) drive organ failure and contribute substantially to patient mortality [2]. Therefore, careful and reasoned fluid resuscitation after traumatic injury represents a crucial step in early posttraumatic treatment to prevent deaths [3]. The clinical value of colloids such as gelatine or hydroxyethyl starch (HES) is still debated in the scientific community and current clinical guidelines do not provide clear recommendations [3–5].

While beneficiary effects of colloids have been proposed based on physiological rationale and confirmed in certain clinical settings [4, 6, 7], both, HES and gelatine solutions are known to impair hemostasis [8–11]. A colloid-induced hypocoagulative state may have substantial consequences after severe tissue trauma and hemorrhage by further driving trauma induced coagulopathy (TIC [12, 13]). On the other hand, extensive fluid administration may aggravate TIC through dilution and acidification [13, 14], rendering colloid administration appealing to reduce the total amount of volume substitution necessary to maintain organ perfusion.

Thromboelastometric measurements have shown that hemodilution with colloids, such as HES or gelatin results in prolonged clot formation and reduced clot firmness [8, 11]. This may, at least in part, be attributed to compromised fibrinogen conversion and weakened fibrinogen-fibrin interaction [9, 10]. Furthermore, a contributive effect of platelet dysfunction has been proposed [11, 15]. Experimental in-vivo models of hemodilution yielded inconclusive results [15–18], but there is a substantial difference in hemostasis physiology between animals and men [19, 20].

We report here, in a recently established murine model of thorax trauma and HS, a higher mortality when mice were resuscitated with Gelafundin compared to crystalline solution. Motivated by these pilot findings, we set out to generate systematic comparative data on the effects of volume dilution with balanced crystalline solution Jonosteril, gelatin solution (Gelafundin 4%) and HES (130/0.42, 6%; Tetraspan) on platelet function and plasmatic hemostasis in a standardized and relevant translational, citrate-/heparin-free human ex vivo whole-blood model.

## Materials and methods

### Murine model of thorax trauma and hemorrhagic shock

Addressing the 3 R-principles (replace–reduce–refine), in-vivo data was generated in a retrospective, exploratory sub-group analysis of pilot experiments in a murine model of thorax trauma (TxT) and hemorrhagic shock (HS; Reg.-No.: 1409). This model, including a detailed protocol and data on vital signs and inflammation parameters in different experimental groups, was previously published [21]. A summary of the protocol can be found in the supplemental materials (Suppl. Methods 1; Suppl. Fig. 1).

### Human ex vivo whole-blood model

**Fig. 1:**
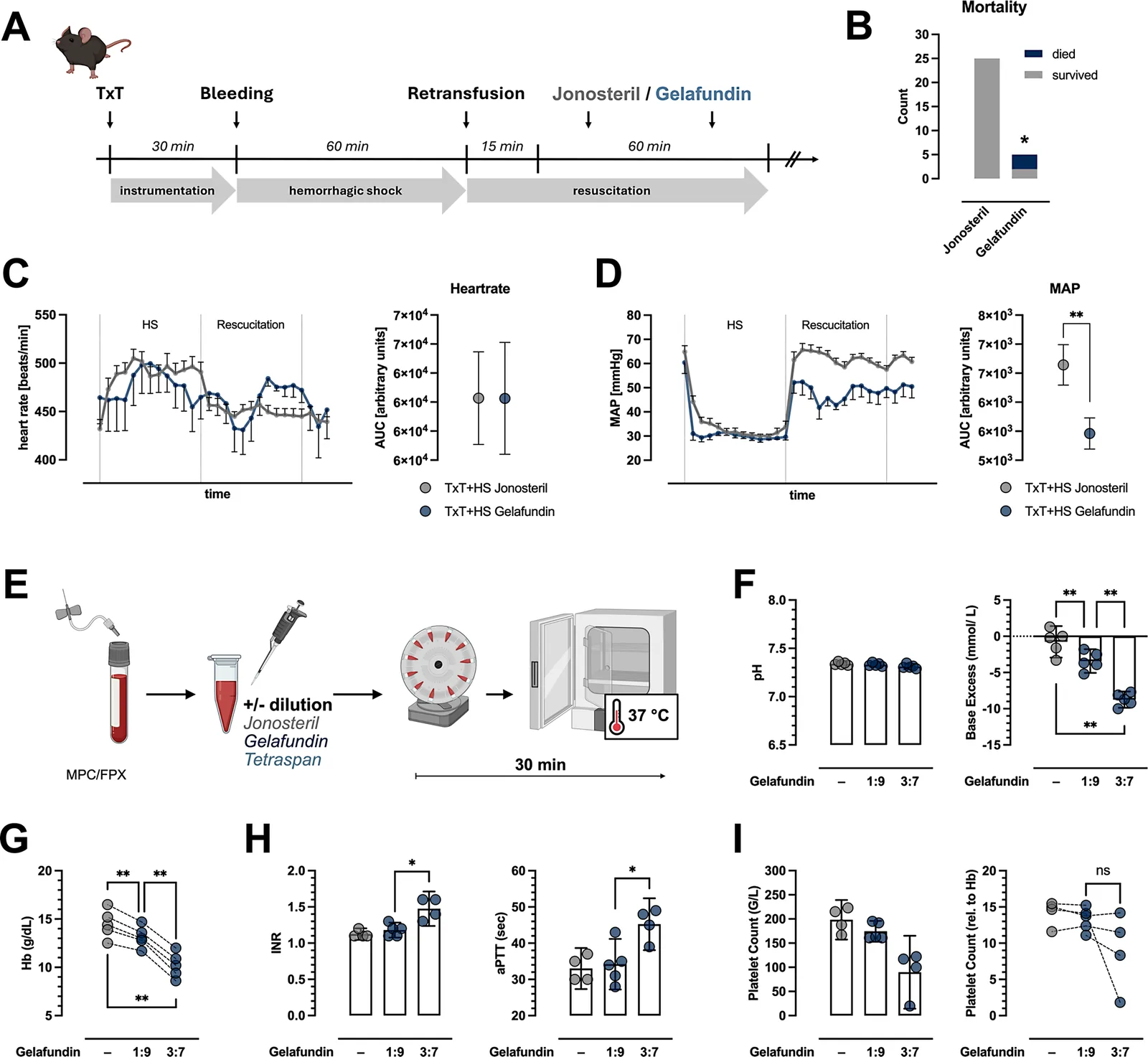
Consequences of volume resuscitation with Gelafundin in a murine model of thorax trauma and hemorrhagic shock. **A**) schematic of the murine model of thorax trauma (TxT) and hemorrhagic shock. **B**) animals resuscitated with 2 × 500 µl of Gelafundin showed significantly higher mortality than animals resuscitated with crystalline solution (Jonosteril). Absolute animal numbers shown, with count of fatal outcome in the Gelafundin group shaded (no fatalities were observed in the Jonosteril group). There was no relevant difference between heart rates of both groups during shock and resuscitation phase (**C**), however the area under the curve (AUC) of the mean arterial pressure (MAP) was significantly smaller in the Gelafundin group (**D**). **E**) schematic of the ex vivo human whole blood (hWBM) used for this study. **F**) in the hWBM, dilution with Gelafundin up to a ratio of 3:7 (300 µl Gelafundin + 700 µl of whole blood) did not change the blood pH after 30 min of incubation, but significantly consumed buffering bases, as visible by a ratio-dependently increasing base deficit (right graph). **G**) as expected, dilution resulted in lower hemoglobin (Hb), altered parameters of plasmatic coagulation (**H**) and lower platelet counts (**I**). Of note, one sample (3:7 dilution) showed macroscopic clotting after 30 min of ex vivo incubation and a profoundly reduced platelet count, even when normalized to Hb values. *p < 0.05; **p < 0.01; ns: not significant. In (**B**), statistical significance was tested via Fisher’s exact test. HF/MAP source data in (**C**) and (**D**) depicted as group mean ± standard error of the mean. For better visibility, statistically non-significant differences were only marked, if of relevance for the experimental hypothesis. INR: international normalized ratio; aPPT: activated partial thromboplastin time; schematics created with *Biorender.com*

To investigate the effects of volume resuscitation with either, *Jonosteril* (Fresenius Kabi, Germany), *Gelafundin* (Braun, Germany; Supp. Tab. 1) or hydroxyethyl starch (HES, 130/0.42; *Tetraspan*; Braun, Germany; Supp. Tab. 1), we used a previously published human ex vivo whole-blood model that allows citrate-free incubation and assessment of hemostatic and immunological functions (hWBM; Fig. 1 E, Suppl. Fig. 2 [14, 22]). The study was approved by the independent ethics committee of the University of Ulm (No. 103/20) and was carried out strictly adhering to the Declaration of Helsinki. Each study proband gave written informed consent before any of the respective experiments were carried out.

In brief, blood from healthy donors (both male and female) was drawn and collected in in neutral monovettes (Sarstedt, Germany) preloaded with fondaparinux (Arixtra®; Aspen Pharma Trading Ltd., Ireland) and immediately transferred to 2-methacryloyloxethyl phosphorylcholine polymer (MPC, Lipidure®; NOF Corporation, Japan) coated 2 ml tubes. Depending on the experimental set-up, crystalloid or colloid solutions were added at respective ratios and samples were incubated at 37 °C for 30 min on a spinning wheel (see Suppl. Methods 2 and Suppl. Fig. 2 for details).

After incubation, samples were further processed for respective analyses (see below).

### ROTEM

Rotational thromboelastometry (ROTEM) was used to assess whole-blood coagulation dynamics. Either directly after volume dilution (termed “baseline”) or following ex vivo incubation for 30 min, a respective volume from each whole blood sample was citrated, kept at room temperature, and measured within 1 h on a ROTEM® delta analyzer (Tem Innovations, Germany). For all assays, blood was first recalcified with star-tem® 20 according to the manufacturer’s instructions.

The extrinsic and intrinsic coagulation pathways were evaluated using EXTEM and INTEM assays, respectively, by addition of the corresponding tissue factor–based (ex-tem) or ellagic acid–based (in-tem) activating reagents. Fibrin-based clot formation in relative isolation from platelet contribution was assessed using the FIBTEM assay, in which platelets are functionally inhibited by cytochalasin D provided with the fib-tem reagent. All respective reagents were purchased from Tem Innovations, Germany.

For each sample, standard ROTEM parameters were recorded, including clotting time (CT), clot formation time (CFT), α-angle, maximum clot firmness (MCF) and time to maximum clot firmness (MCFt), where applicable.

### Blood gas analysis, blood count, ELISA and flowcytometry

For blood gas analysis, 100 µl of whole-blood from incubated samples were collected in heparin-coated capillaries and measured with a ABL800 flex analyzer (Radiometer Medical ApS, Germany). Blood count, Quick-values, INR (international normalized ratio) and aPTT (activated partial thromboplastin time) were determined by the certified routine laboratory of the Ulm University Medical Center.

We analyzed sample concentrations of thrombin-antithrombin complexes (TAT), von Willebrand factor (vWF), sCD154 (=CD40L), anaphylatoxins C3a and C5a, and soluble membrane attack complex (sC5b-9) via commercially available ELISA kits (see Suppl. Methods 3 for details).

To assess the activation state and activation capacity of platelets from diluted samples, CD62P and CD63 surface expression was analyzed from whole-blood samples via flowcytometry (see Suppl. Methods 4 for details).

### Statistics

All data were analyzed and visualized with Prism 10 (GraphPad Software, USA). Data sets were tested for possible outliers where applicable, using the ROUT method with a maximum false discovery rate of 5%. Removed outliers are referred to in the respective figure legends. All data sets were visualized as single values, group mean and 95% confidence interval, unless stated otherwise in the respective figure legends.

Differences between group means were tested for statistical significance using repeated measures (RM) one-way ANOVA/Prism 10 mixed-effects model and applicable post-hoc tests with correction for multiple comparisons. Analyses were corrected for inequality of variances or missing sphericity. Categorical data was analyzed for statistical significance using the Fisher’s exact test.

Results with *p*-values < 0.05 were considered statistically significant. The level of significance was depicted as follows: *p < 0.05; **p < 0.01; ns: not significant. For better visibility, statistically non-significant differences were only marked, if of relevance for the experimental hypothesis.

## Results

### Volume resuscitation with Gelafundin is associated with poor outcome in a murine model of TxT and HS

In part, the overarching hypothesis for the present study originated from the incidental observation, that volume resuscitation with the colloid solution Gelafundin was associated with poor pressure response during resuscitation and increased mortality in a murine model of TxT and HS. We retrospectively analyzed mortality, heart rate and mean arterial pressure (MAP) in a subgroup of mice that received Gelafundin for volume resuscitation, and compared these data with those from mice resuscitated with Jonosteril in a previously published murine study [21]. We observed unexpectedly high mortality in the experiment subgroup resuscitated with Gelafundin (Fig. 1B). In contrast, no fatal outcome occurred among animals resuscitated with crystalline solution after pressure-controlled HS. While heart rates were comparable between both analyzed subgroups (Fig. 1C), mice infused with Gelafundin showed lower MAP values throughout resuscitation phase resulting in a significantly smaller group area under the curve (AUC) for the MAP (Fig. 1D).

### Dilution with Gelafundin leads to a significant base deficit in an ex vivo whole-blood model of volume resuscitation

To further investigate the effects of substantial volume resuscitation with colloidal solutions, we established a clinically relevant ex vivo model of volume dilution of human whole-blood, based on previously published models from our group (Fig. 1E[14, 22]). We tested increasing ratios of dilution to a maximum of 3:7, corresponding to a clinically relevant volume replacement of 1.5–2 L in an average weighing adult.

Base excess dropped ratio-dependently to maximum deficit of 8.5 mmol/L, while pH remained stable, reflecting sufficient buffering capabilities of whole-blood in our model (Fig. 1F). Hemoglobin levels correlated well with the dilution ratio (Fig. 1G). We did not observe any relevant influence of Gelafundin on global parameters of plasmatic hemostasis or platelet counts, beyond the effects of dilution (Fig. 1H,I). Of note, at the highest dilution ratio, we observed occasional macroscopic clotting of the samples after ex vivo incubation (Fig. 1I, right panel, lowest platelet value).

### High ratios of volume dilution with colloids result in macroscopic clotting ex vivo and increased levels of platelet-derived von Willebrand factor

The main goal of this study was to determine the effects of different resuscitation regimens using colloidal volume expanders compared with a crystalloid solution on plasmatic hemostasis and platelet function in a well-established ex vivo hWBM. We compared volume dilution with either Gelafundin (40 mg/mL; 4%) or hydroxyethyl starch (HES; Tetraspan 60 mg/mL; 6%).

**Fig. 2:**
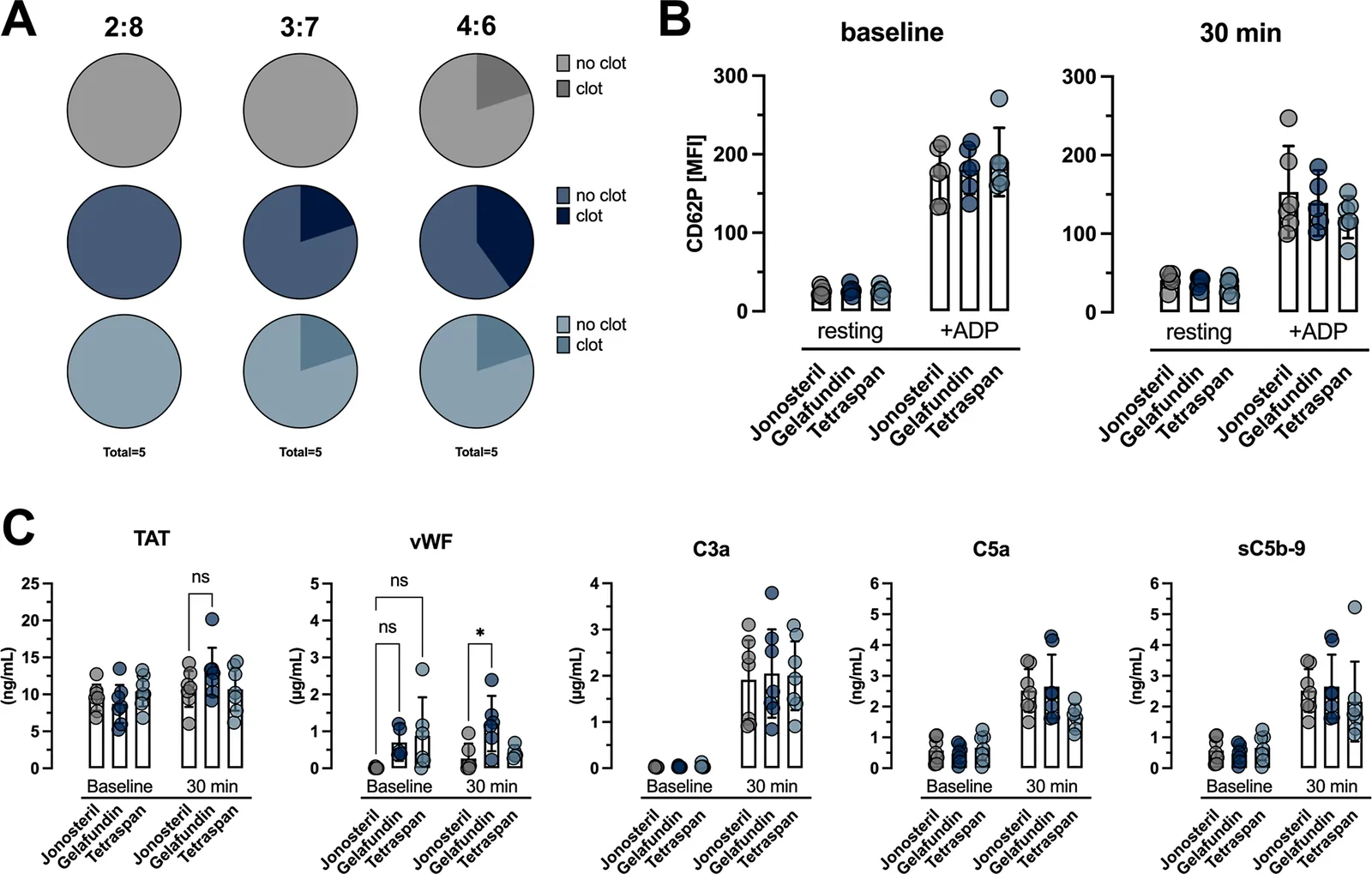
Prothrombotic effects of Gelafundin and Tetraspan. **A**) fraction of macroscopically clotted samples after incubation for 30 min ex vivo at different dilution ratios (2:8–3:7–4:6). Samples diluted with colloids showed a tendency to clot early and more frequently than samples diluted with crystalline solution. **B**) dilution of whole blood ex vivo did not activate platelets, nor did it impair platelet activation capacity to 5 µM ADP by means of surface CD62P expression. **C**) thrombin-antithrombin (TAT) complex formation was comparable among groups. However, samples diluted with colloids showed a tendency for increased release of von Willebrand-factor (vWF; from platelets) with a significant raise in vWF levels after 30 min incubation with gelafundin dilution at a ratio of 3:7 compared to crystalline solution. No relevant differences in complement activation were observed. All data in (**B**) and (**C**) From a dilution ratio of 3:7. *p < 0.05; ns: not significant. 3 outliers removed from vWF data set (Jonosteril, baseline: 1.4 µg/mL; Gelafundin, baseline: 4.1 µg/mL; Tetraspan, 30 min: 2.9 µg/mL); 1 outlier removed from C5a data set (Tetraspan, 30 min: 5.2 ng/mL). For better visibility, statistically non-significant differences were only marked, if of relevance for the experimental hypothesis. sMAC: soluble membrane attack complex; MFI: mean fluorescent intensity

Based on our observations of higher mortality in the murine model and occasional ex vivo clotting at higher dilution ratios, we documented macroscopic clot formation over 30 min of incubation under increasing dilution with Jonosteril, Gelafundin, or Tetraspan (Fig. 2A). We observed a trend towards more frequent clot formation and a lower dilution threshold with colloid solutions; however, these observations did not reach statistical significance. Notably, modelled resuscitation with both Gelafundin and Tetraspan was accompanied by significantly lower pH and a more pronounced base deficit compared with Jonosteril (Suppl. Table 2).

Dilution at a ratio of 3:7 did not result in immediate platelet activation or in a relevant change in platelet activation capacity, as assessed by surface CD62P (alpha granule derived) and CD63 (dense granule derived) expression (Fig. 2B, Suppl. Fig 3B). However, Gelafundin dilution at the same ratio significantly increased vWF plasma levels after 30 min of ex vivo incubation and showed a strong trend towards higher levels already at baseline (*p* = 0.063; Fig. 2C). In our model, which lacks endothelial cells, vWF is exclusively platelet derived. In contrast, no relevant release of sCD154 as another marker of platelet activation was obtainable (Suppl. Fig 3A). We did not observe relevant differences in TAT formation or complement activation among the experimental conditions.

### Gelafundin and tetraspan induce a hypocoagulable ROTEM profile

At a dilution ratio of 3:7, global viscoelastic hemostasis was assessed by ROTEM at baseline (immediately after dilution) and after 30 min of ex vivo incubation using EXTEM, INTEM and FIBTEM (with platelet inhibition).

**Fig. 3:**
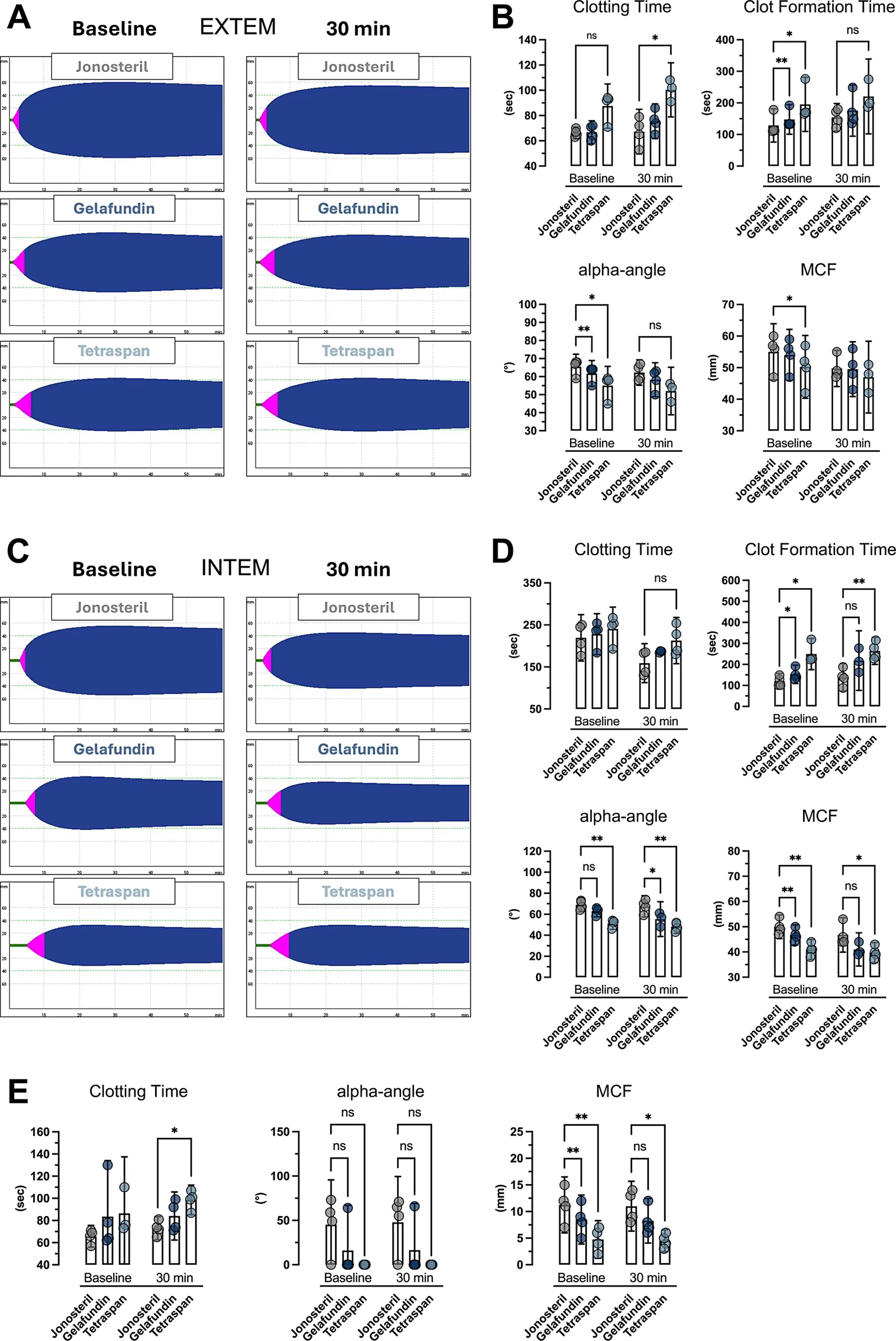
Differential impact of colloids on rotational thromboelastometry (ROTEM). For viscoelastic assessment, 300 µl of either crystalline solution (jonosteril), Gelafundin or hydroxyethyl starch solution (tetraspan) were added to 700 µl of whole blood to yield a dilution ration of 3:7. ROTEM analysis was performed both directly after dilution (baseline) and after 30 min of ex vivo incubation. **A**) representative EXTEM-graphs from all experimental groups visualizing compromised clot formation for colloid-diluted samples. **B**) EXTEM read-outs demonstrated diminished clotting capacity of the external pathway by means of clot formation and firmness for colloid-resuscitation. **C**) representative INTEM-graphs from all experimental groups visualizing impaired clot formation for colloid-diluted samples. **D**) as for EXTEM read-outs, INTEM parameters showed a significant reduction of clot formation capacities. **E**) FIBTEM analysis showed a similar pattern as EXTEM and INTEM. *p < 0.05; **p < 0.01; ns: not significant. For better visibility, statistically non-significant differences were only marked, if of relevance for the experimental hypothesis. MCF: maximum clot firmness

Dilution with Gelafundin or Tetraspan resulted in a clearly hypocoagulable viscoelastic profile compared with Jonosteril across all three assays. Impairment of clot initiation and propagation, as well as reduced clot firmness, was already evident at baseline, with only marginal additional changes after 30 min of incubation (Fig. 3A,C).

The effect was most pronounced with Tetraspan, which showed prolonged clotting time (CT) and clot formation time (CFT), together with a reduced α-angle (alpha) and maximum clot firmness (MCF) in both EXTEM and INTEM, indicating delayed clot initiation, impaired clot build-up and decreased clot stability (Fig. 3B,D). Gelafundin dilution revealed a broadly similar hypocoagulable pattern, albeit less marked and less consistent across individual parameters and ROTEM assays.

In notable contrast to the ROTEM findings, routine parameters of global hemostasis (Quick, INR and aPTT) were largely unaltered (Suppl. Tab. 2)

### Gelafundin and Tetraspan drive non-linear, ratio-dependent impairment of clot formation

**Fig. 4:**
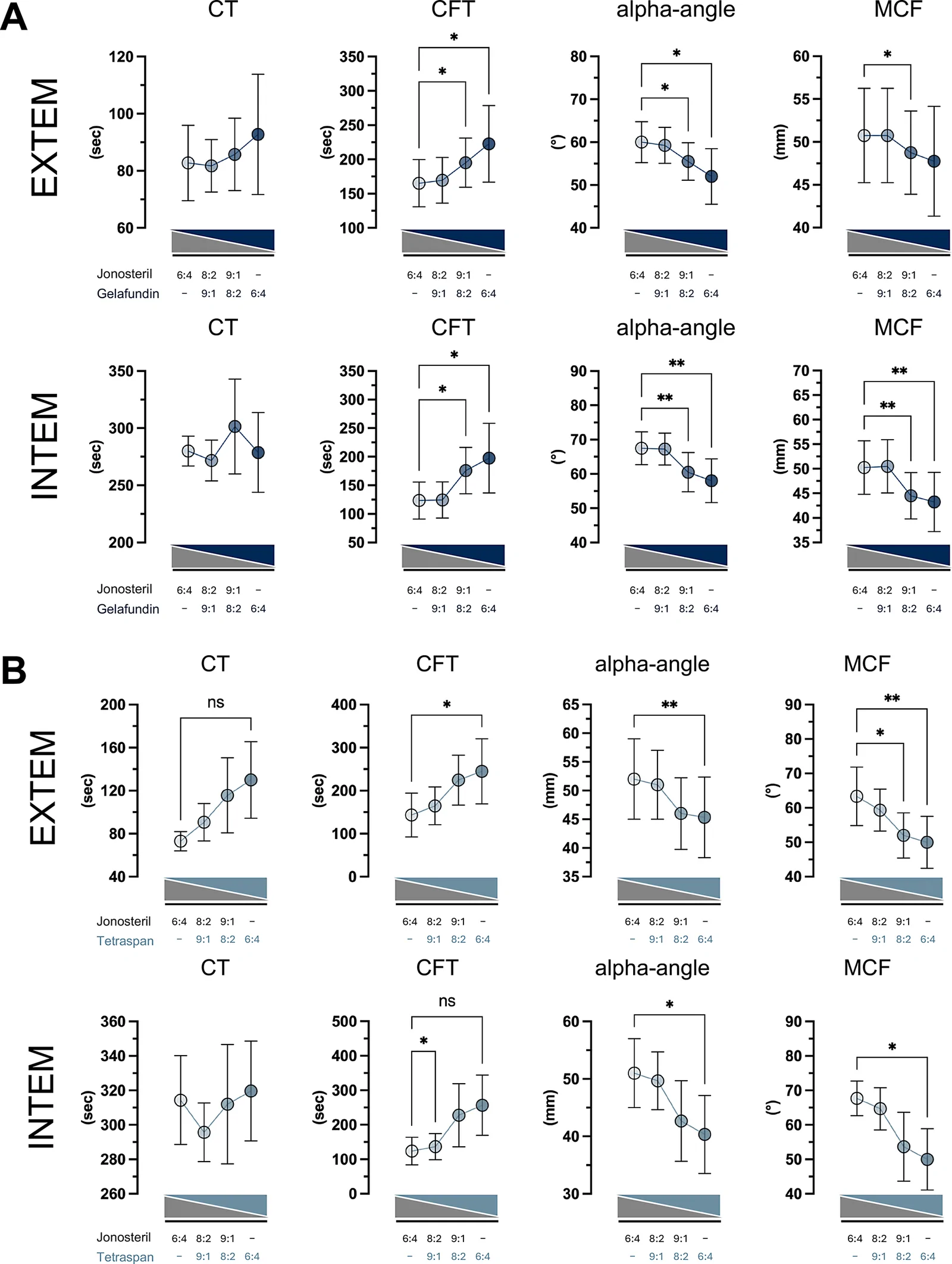
Effects of different combinations of crystalline and colloid solutions on clot formation. Human whole blood was diluted with different a combination of crystalline solution (Jonosteril) and colloids (A: Gelafundin; B: tetraspan) at different ratios and clot formation was analyzed with ROTEM. Impairment of clot formation was mainly induced by colloid dilution with similar patterns for Gelafundin (**A**) and Tetraspan (**B**). See text for further details. *p < 0.05; **p < 0.01; ns: not significant. Data depicted as group mean ± standard deviation. For better visibility, statistically non-significant differences were only marked, if of relevance for the experimental hypothesis. CT: clotting time: CFT: clot formation time; MCF: maximum clot firmness

To further improve the translatability of our ex vivo resuscitation model and to dissect potential additive effects, we next combined Jonosteril with either colloid while keeping the overall blood:diluent ratio constant at 3:7. Whole-blood was diluted with mixtures containing increasing fractions of Gelafundin or Tetraspan and EXTEM and INTEM were measured immediately after dilution (Fig. 4A,B).

Across both assays, we observed a hypocoagulable viscoelastic profile with a nearly sigmoidal dependence on the crystalline:colloid ratio. This pattern suggests an intrinsic resilience of whole-blood to modest colloid fractions, followed by a tipping point beyond which clot initiation, propagation and firmness are markedly impaired. Increasing colloid content was associated with progressive prolongation of CFT and reduction in MCF and α-angle, indicating slower clot build-up and reduced clot stability.

Consistent with our single-fluid experiments, these effects were more pronounced when Jonosteril was combined with Tetraspan (Fig. 4B) than with Gelafundin (Fig. 4A). Taken together, these findings support that the observed ROTEM alterations reflect inherent effects of colloid volume expanders themselves, rather than mere consequences of volumetric dilution.

## Discussion

Hemorrhage and ensuing circulatory shock substantially drive coagulopathy and multi-organ damage, and thereby increase mortality after sever tissue trauma [2, 12, 23]. Rational fluid resuscitation still remains one of the hallmarks of early shock management, however, the right choice of fluid is still under debate [3]. Moreover, hemodilution through aggressive volume replacement with crystalline solutions is associated with multi-organ failure and over-all morbidity in trauma patients [24]. Colloid solutions have been demonstrated to have a more favorable hemodynamic effect per transfused volume [25], thus potentially reducing the total amount of volume required to achieve hemodynamic stability. This in turn may mitigate the additional impact of iatrogenic dilution on TIC development and course.

Tissue trauma and especially trauma-associated hemorrhage and shock lead to a disintegration and shedding of the endothelial glycocalyx layer, which in turn may diminish the retaining effect of colloids on the intravascular system and additionally enhance their hypocoagulative effects [2, 6]. Notably, a protective effect of HES 130 on the integrity of the glycocalyx layer has been shown in murine and rat models of acute hemorrhage [26, 27]. However, colloids negatively affect clot formation and stability [8], making them a somewhat Janus-faced choice for volume resuscitation. Thus, the inconclusive evidence surrounding different volume resuscitation regimens – as captured in meta-analyses primarily reporting mortality outcomes – may reflect opposing mechanisms, with beneficial intravascular volume expansion counterbalanced by deleterious effects on hemostasis.

With this study we present comparative data regarding the effects of polysuccinylated gelatin (Gelafundin 4%) and HES 130/0.42 (Tetraspan, 6%) on hemostasis and platelet function ex vivo. We used the crystalline solution Jonosteril as a control to estimate the differential effects of gelatin and HES beyond dilution. Balanced crystalline solutions are generally recommended as the first line of volume resuscitation for hypotension after trauma [3].

During the establishment phase of a murine model of TxT and HS, we observed, that volume resuscitation with Gelafundin lead to unexpectedly high mortality and retrospective analysis comparing the vital parameters of the experimental sub-groups showed a significantly lower AUC of the mean arterial pressure during resuscitation phase. Upon examination we noticed substantial blood clotting in the heart and stem vessels (data not shown). These findings are in line with recently published data from a rat model of hemodilution, where HES resulted in a more procoagulant thromboelastometric profile, underlining the limited comparability of animal models with human physiology [16]. It is important to note, that an anaphylactic reaction to Gelafundin cannot be formally excluded as a contributing factor to the increased mortality observed in the murine trauma–hemorrhage model. However, in classical murine models of systemic anaphylaxis, this typically requires prior passive or active sensitization [28], and no deliberate gelatin sensitization was performed in our experimental setting. Moreover, the occurrence of such a reaction in several naïve laboratory mice within the same cohort would appear less likely than a fluid-associated physiological effect, although this cannot be resolved retrospectively. Future studies should therefore include dedicated monitoring for anaphylaxis-associated features, such as acute hypothermia, abrupt hemodynamic collapse and mast-cell activation markers, to clearly distinguish allergic from hemostatic or hemodynamic mechanisms.

Based on a recently published ex vivo protocol to model TIC in human whole-blood [14], we established an experimental set-up to investigate the effects of various ratios of hemodilution with crystalline and colloid fluids. In our model, volume substitution with colloids did not directly affect CD62P/CD63 expression on platelets nor their reaction capacity. The effect of colloids, especially HES on platelet activation has not yet been conclusively determined. In-vivo analyses after infusion of small volumes of HES (of different molecular weights) in children did not result in relevant changes in CD62P expression [29]. In contrast, high-ratio hemodilution in-vitro with either HES 130/0.4 or HES 200/0.5 did result in a small increase in the CD62P^+^ platelet population after ADP stimulation but conversely impaired platelet activation capacity to thrombin receptor-activating peptide [30]. The significant effects of dilution with both gelatin and HES in our FIBTEM assays – i.e. without platelet contribution to clot formation – suggests that any platelet dysfunction may be negligible.

We observed that dilution with colloid solutions – especially with Gelafundin – triggered vWF release, which in our model may serve as a surrogate marker of platelet activation [14]. This could reflect a colloid-induced pro-coagulatory phenotype that may explain the slightly higher incidence of macroscopic clotting in colloid-diluted blood. However, the isolated increase in plasma vWF should be interpreted with caution, as it contrasts with the absence of changes in platelet surface activation markers. Although platelet vWF is stored in α-granules and can be released upon platelet activation, CD62P and plasma vWF do not necessarily represent identical readouts: CD62P reflects α-granule membrane translocation, whereas plasma vWF reflects release of soluble cargo, and differential packaging and secretion of platelet α-granule cargo have been described [31, 32]. Moreover, flow cytometry-based analyses in this model can only include cells in suspension and exclude platelets already trapped within (micro-)clots ex vivo. Therefore, they might not fully reflect the activation status of all platelets present in the sample, whereas vWF – once released – remains detectable in plasma. Thus, a discordant pattern may be biologically possible.

At the same time, the lack of concordant changes in CD62P, CD63, and sCD154 argues against overt global platelet activation as the dominant explanation in our model; this interpretation is further supported by the persistent platelet-independent abnormalities in FIBTEM. In addition, technical interference of colloid formulations with analytical assays cannot be fully excluded, as synthetic colloids have been shown to interfere with laboratory measurements, including protein assays and ELISA-based biomarker detection [33, 34]. We therefore regard the vWF finding as an isolated and hypothesis-generating signal rather than definitive evidence of broad platelet activation. Furthermore, no differences in TAT formation or complement activation were detected between the experimental groups, suggesting that major effects on blood serine-protease cascades and fluid-phase immunity are unlikely.

When we diluted human whole-blood at a ratio of 3:7 (i.e. crystalloid/colloid:whole blood) – which is comparable to substantial volume replacement and hemodilution, as seen in trauma patients with hemorrhagic shock – resulted in a hypocoagulable ROTEM profile with prolonged clot formation and reduced clot stability. In all three ROTEM assays (i.e. EXTEM, INTEM and FIBTEM) and almost each parameter, Tetraspan revealed a more profound effect on the viscoelastic profile, than Gelafundin. The CT, which reflects the initial thrombin generation was prolonged by hemodilution with Tetraspan across all assays, reaching statistical significance after incubation over 30 min. This observation supports our hypothesis, that the interfering effect of HES on the plasmatic coagulation may be time- (or rather exposure-) dependent. In contrast, Gelafundin did not seem to have a relevant effect on clot initiation but rather impair propagation – supposedly by interfering with fibrinogen polymerization and conversion. This is in line with previous ROTEM studies investigating the effects of gelatin, where the effect on the CFT was most pronounced and least reversible by single factor or fresh frozen plasma substitution [9]. In terms of monitoring clot propagation, the alpha angle proved more reliable in our model with better reproducibility and less variance. Whether this is a technical artefact, and whether this prompts respective recommendations for diagnostics and patient monitoring needs to be further evaluated in a clinical setting. Hemodilution with gelatin formulations was shown to result in a less dense fibrin-mesh [35]. In our model, impairment of fibrin polymerization and resulting reduced strength of the forming clot is evident regarding the significantly reduced MCF values, especially in the FIBTEM assay. However, additional assessment of fibrin monomers and D-dimers would have provided valuable complementary information on fibrin formation and turnover and may have helped to further contextualize this ROTEM/FIBTEM findings.

Of note, incubation over 30 min ex vivo did not further enhance the effects on the viscoelastic profile we noticed in baseline measurements. This supports the hypothesis of a stable molecular interaction of colloids with coagulation factors, rather than an active (catalytic) effect. An important limitation to our study is the closed-loop set up, that neglects the impact of the endothelium and of pharmacokinetics (i.e. pharmacon metabolism and elimination) that would occur in-vivo. However, reported plasma half-lives of both gelatin and HES exceed the time-frame usually relevant for early trauma management – especially when repeated infusion is considered [36, 37]. The discrepancy between largely unchanged INR/aPTT and the pronounced hypocoagulable ROTEM profile likely reflects the different biological information captured by these assays: whereas routine coagulation tests are plasma-based and primarily assess initial fibrin formation, viscoelastic assays evaluate whole-blood clot initiation, propagation, firmness and lysis and are therefore more sensitive to functional impairment of clot formation, including impaired fibrin polymerisation after colloid hemodilution [38].

Mixing Jonosteril with colloid at increasing fractions produced a clear, ratio-dependent deterioration of ROTEM profiles that mirrored the data from the preceding experiments. As with single fluid dilution, the observed pattern was more pronounced when Jonosteril was combined with Tetraspan, where even modest colloid fractions already reduced MCF and alpha-angle, while higher fractions caused a marked hypocoagulable shift. Gelafundin showed the same direction of effect but with smaller amplitude and higher inter-experimental variance. Overall, these data propose a non-linear, ratio-dependent impact of colloid admixture on clot formation and support the idea of a pharmacodynamic “tipping-point”, where the influence of gelatin and HES become substantially detrimental. It is tempting to speculate, that the fluid debate may not be a simple matter of “crystalloid versus colloid”, but rather of how much of each the coagulation system can tolerate and where the additive effect of colloid resuscitation may be beneficial for patient outcome. Defining these ratio-dependent tipping points may be relevant for hemostatic safety in a clinical setting and warrants further research both in the laboratory setting and through clinical trials. We acknowledge that, in contemporary trauma care, blood products and hemostatic resuscitation strategies have largely displaced synthetic colloids from first-line use. Nevertheless, colloids remain discussed as restricted second-line or rescue options in current guidelines [3], which preserves the relevance of mechanistic data on their hemostatic effects.

Our study and the models used have inherent limitations that must be considered when interpreting the data. The apparent contrast between our retrospective murine observations and the hypocoagulable ROTEM profile in the ex vivo human whole-blood model should be interpreted with caution. The murine findings were not generated in a systematic hemostasis-focused study, but rather represented an exploratory retrospective observation that motivated the present work; accordingly, this study lacks comprehensive data on hemostasis and platelet function and additional confounding factors cannot be excluded. In addition, murine and human hemostasis differ substantially, and the in vivo trauma setting includes multiple additional determinants of coagulation, including endothelial and organ-derived signals, which are not captured by our ex vivo model. Against this background, the murine and ex vivo findings should not be viewed as directly contradictory, but rather as reflecting different biological contexts and levels of analysis.

Stable incubation ex vivo requires (low-dose) factor Xa inhibition and artificial surface protection, and therefore cannot fully mirror the dynamic in vivo situation. Moreover, our model does not reproduce the influence of endothelial cells and remote organs on hemostasis and inflammation. Nevertheless, this model was previously validated to maintain experimentally relevant hemostatic and inflammatory reactivity while preventing unspecific ex vivo microclotting [14, 22, 39].

The present study is primarily descriptive and does not establish a definitive molecular mechanism by which gelatin or HES impair hemostasis. By providing a systematic and comparatively comprehensive functional characterisation of the hemostatic effects of balanced colloids in a controlled human whole-blood model, it still offers a relevant framework for future studies to dissect the precise pathways by which colloids affect coagulation and platelet biology.

## Conclusion

In a human ex vivo whole-blood model, we show that balanced colloid solutions, particularly HES 130/0.42, induce a marked hypocoagulable ROTEM profile, whereas routine coagulation tests remain largely unchanged. Gelatin and HES predominantly impair clot propagation and firmness, with FIBTEM indicating a dominant effect on fibrin(ogen)-dependent clot stability rather than overt platelet or serine-protease cascade activation. When colloids are combined with a balanced crystalloid at a fixed dilution, these changes occur in a non-linear, ratio-dependent fashion, suggesting thresholds beyond which clot formation is critically compromised. Together, these findings offer a plausible functional explanation for the ambiguous clinical data on colloid resuscitation and highlight that hemostatic safety depends not only on the fluid chosen but also on the proportion of colloid used.

## Electronic supplementary material

Below is the link to the electronic supplementary material.

Supplementary Material 1

## Data Availability

The datasets used and/or analyzed during the current study are available from the corresponding author on reasonable request.
