# Supplementary material for: Gelatin 4% and hydroxyethyl starch (130/0.42) impair clot formation in a human ex vivo whole-blood resuscitation model
Source: Scand J Trauma Resusc Emerg Med. 2026 Sep 14;34:151. doi: 10.1186/s13049-026-01690-6 (PMC13579869; doi:10.1186/s13049-026-01690-6)
Supplement: Supplementary file 1 [file 13049_2026_1690_MOESM1_ESM.pdf]

Gelatin 4% and hydroxyethyl starch (130/0.42) impair clot formation in a human ex vivo whole-blood resuscitation model

Marco Mannes[1], Doreen Tabea Spiegelburg[1], Thomas Datzmann[1], Anke Schultze[1], Andreas Bauer[2], Gerhard Achatz[2], Markus Huber-Lang[1], Christian Karl Braun[3,4]

[1] Institute of Clinical and Experimental Trauma Immunology, Ulm University Medical Center, Ulm, Germany

[2] Department of Trauma Surgery and Orthopaedics, Reconstructive and Septic Surgery, Sportstraumatology, Trauma Surgery Research Group, German Armed Forces Hospital, Ulm, Germany

[3] Department of Pediatrics and Adolescent Medicine, Ulm University Medical Center, Ulm, Germany

[4] German Center for Child and Adolescent Health (DZKJ), Partner Site Ulm, Ulm, Germany

Supplemental Data

Suppl. Tab. 1
Constituents and chemical properties of the volume solutions used in this study as indicated in the manufacturer's leaflet.

*: for pH adjustment.

	Jonosteril	Gelafundin 4%	Tetraspan 6%
	**Colloid Osmotic Active Substance**		
**Colloid type**	*None* (crystalloid)	Gelatin polysuccinate (modified fluid gelatin)	Hydroxyethyl starch (HES; MW 130 kDa)
**Colloid concentration (g/L)**		40.0	60.0
	**Constituents**		
**Sodium chloride (g/L)**	6.430	5.55	6.25
**Sodium acetate trihydrate (g/L)**	3.674	3.27	3.27
**Potassium acetate (g/L)**	0.393	-	-
**Potassium chloride (g/L)**	-	0.30	0.30
**Magnesium acetate tetrahydrate (g/L)**	0.268	-	-
**Magnesium chloride hexahydrate (g/L)**	-	0.20	0.20
**Calcium acetate (g/L)**	0.261	-	-
**Calcium chloride dihydrate (g/L)**	-	0.15	0.37
**L-malic acid (g/L)**	-	-	0.67
**Other excipients**	Hydrochloric acid*<br>sodium hydroxide*<br>water for injections	Sodium hydroxide*<br>hydrochloric acid*<br>water for injections	Sodium hydroxide*<br>water for injections
	**Electrolyte Molarity**		
**Sodium (Na^+) (mmol/L)**	137	151	140
**Potassium (K^+) (mmol/L)**	4.0	4.0	4.0
**Calcium (Ca^{2+}) (mmol/L)**	1.65	1.0	2.5
**Magnesium (Mg^{2+}) (mmol/L)**	1.25	1.0	1.0
**Chloride (Cl^-) (mmol/L)**	110	103	118
**Acetate (mmol/L)**	36.8	24	24
**L-malate (mmol/L)**	-		5.0
	**Chemical Properties**		
**pH**	5.0–7.0	7.4 ± 0.3	5.6–6.4
**Theoretical osmolarity (mOsm/L)**	291	284	296
**Titratable acidity**	1.0–10.0 mmol NaOH/L	*n.a.*	< 2.0 mmol/L (titration to pH 7.4)

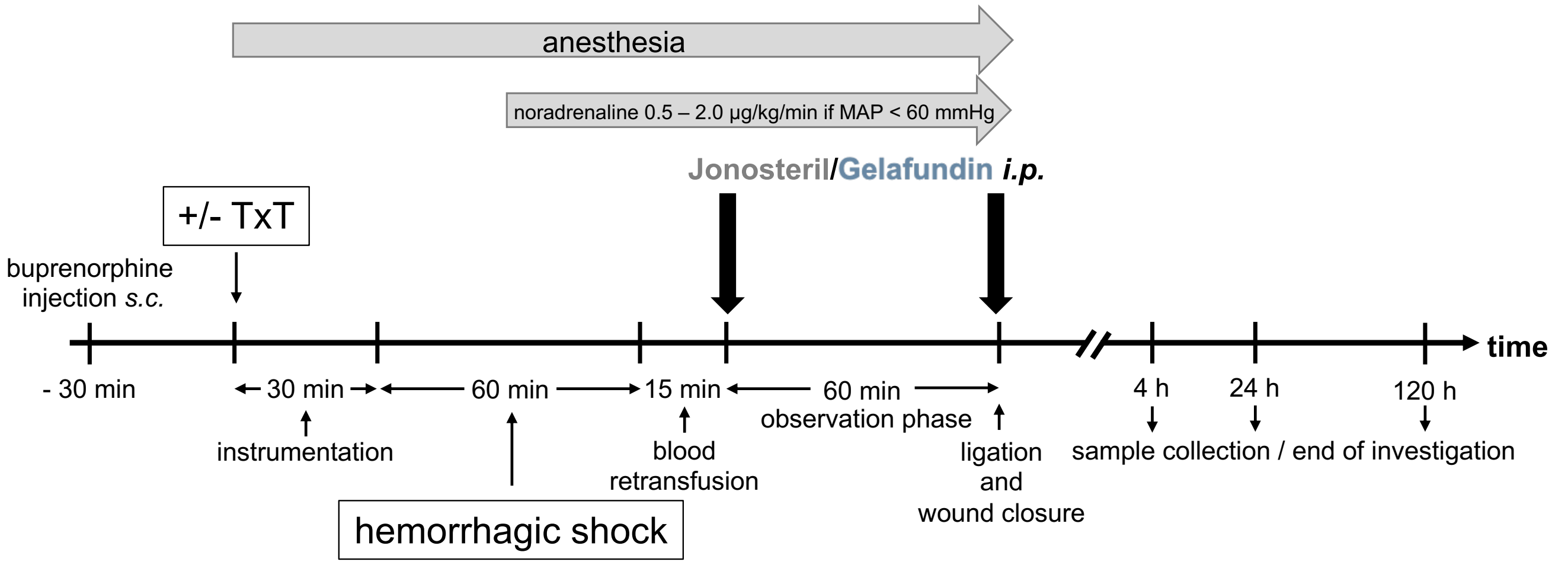

Suppl. Fig. 1
Detailed schematic of the original murine model of thorax trauma (TxT) and hemorrhagic shock referenced in this study.

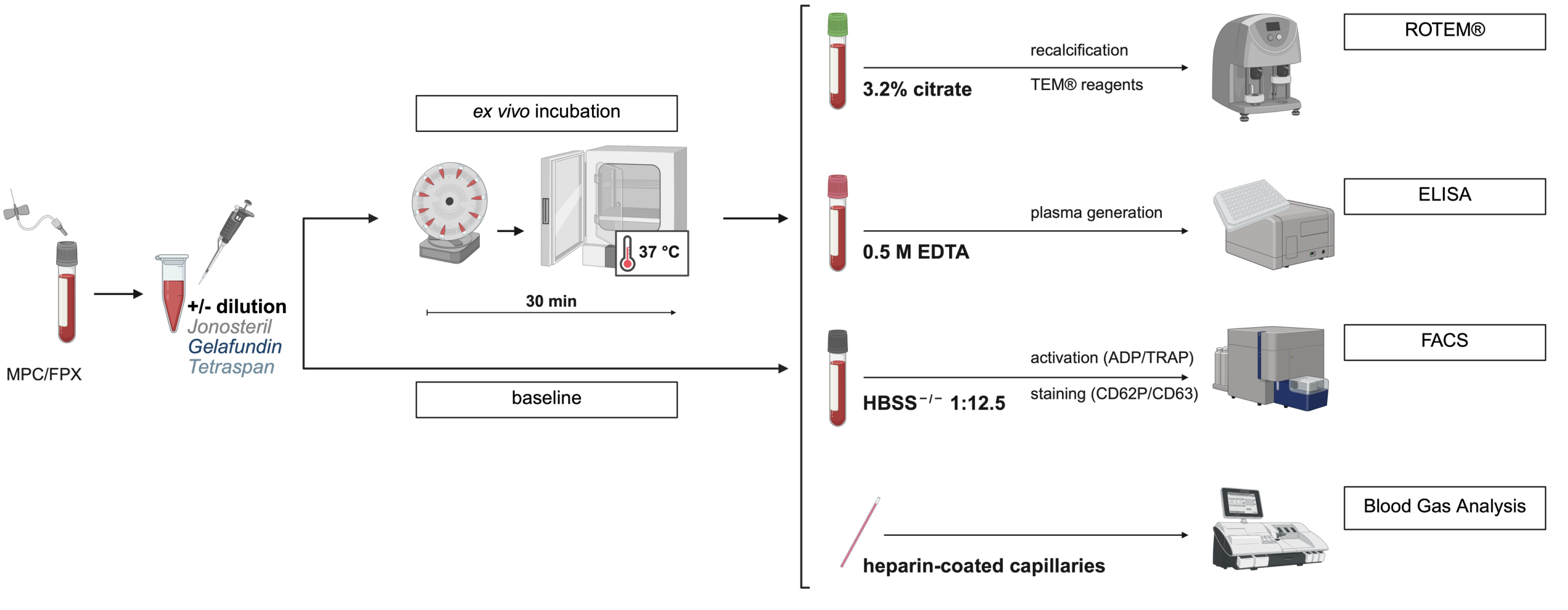

Suppl. Fig. 2
Detailed schematic of the *ex vivo* whole blood model and respective read-out analyses.

MPC: 2-methacryloyloxethyl phosphorylcholine polymer; FPX: fondaparinux; EDTA: ethylenediaminetetra-acetic acid; ADP: adenosine diphosphate; TRAP: thrombin receptor activator peptide;

Image generated with *BioRender.com*.

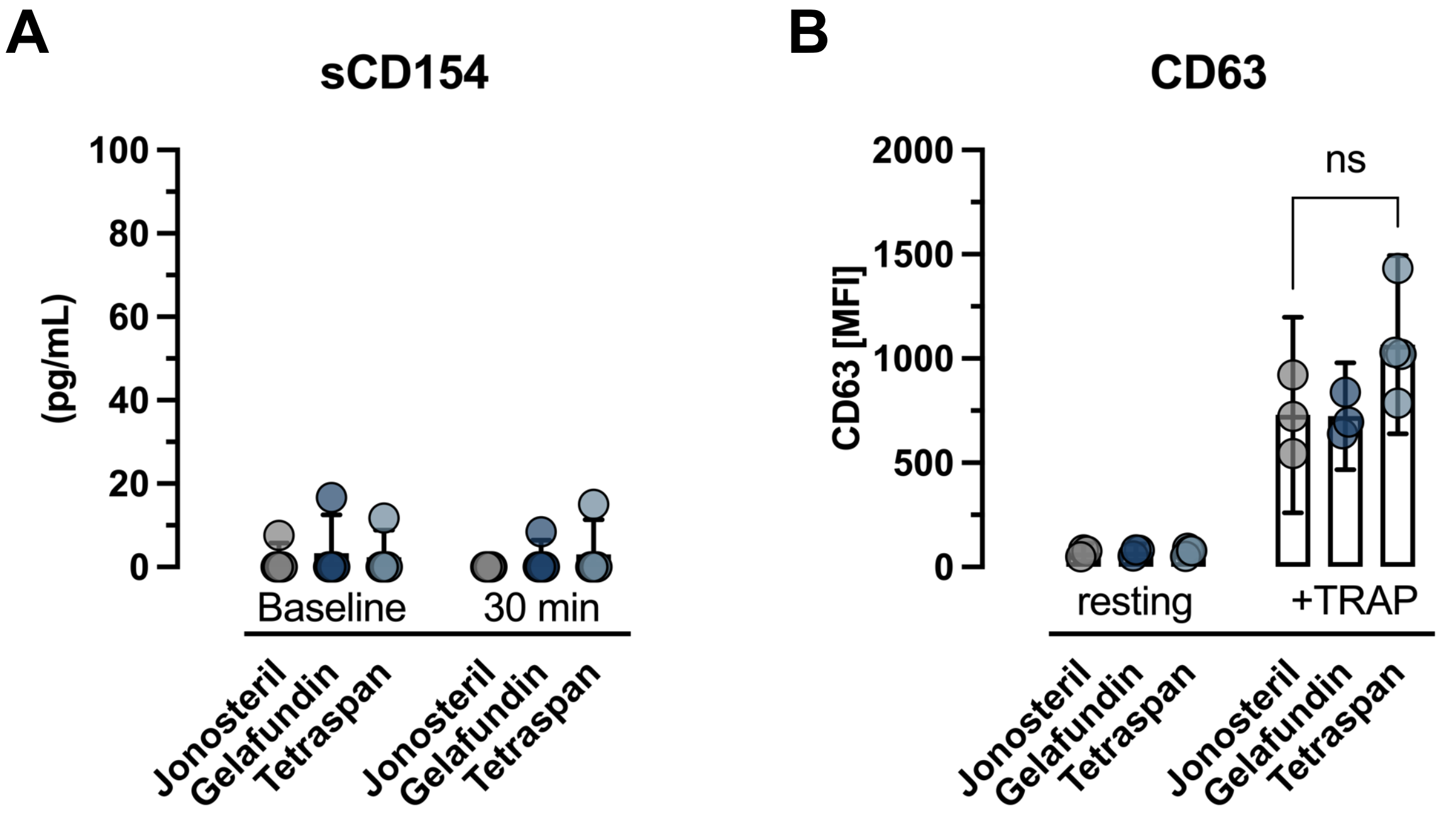

Suppl. Fig. 3

A) Measurement of sCD154 (= sCD40L) in plasma as an additional marker of platelet activation at baseline and after 30 min of ex vivo incubation via ELISA showed no relevant release under any experimental condition. B) Analysis of surface CD63 expression on platelets in whole blood after 30 min of ex vivo incubation showed no difference between experimental groups. Additional stimulation with TRAP (thrombin receptor activating peptide) revealed preserved reaction capacity of platelets with a tendency towards higher expression levels after dilution with Tetraspan.

MFI: mean fluorescence intensity;

Suppl. Tab. 2
Acid-base status, small blood count and routine coagulation parameters at baseline and after 30 min of incubation ex vivo. Samples were generated from the same experiments as shown in Fig. 3 of the main manuscript.

*: $p<0.05$ vs Jonosteril at the same time-point.

blood gas analysis	baseline			30 min		
**parameter**	**Jonosteril**	**Gelafundin**	**Tetraspan**	**Jonosteril**	**Gelafundin**	**Tetraspan**
**pH**	7.382 (± 0.039)	7.362 (± 0.041)	7.36 (± 0.029)	7.343 (± 0.046)	7.320* (± 0.041)	7.320* (± 0.041)
**cBase [mmol/L]**	-8.80 (± 1.83)	-8.07 (± 2.31)	-8.12 (± 2.15)	-9.95 (± 1.77)	-8.95* (± 2.00)	-9.10* (± 1.98)
**$cHCO_3^-$ [mmol/L]**	17.23 (± 0.96)	17.58 (± 1.15)	17.55 (± 1.06)	16.53 (± 0.93)	17.03 (± 0.99)	16.93 (± 0.99)

small blood count	baseline			30 min		
**parameter**	**Jonosteril**	**Gelafundin**	**Tetraspan**	**Jonosteril**	**Gelafundin**	**Tetraspan**
**erythrocytes [Tera/L]**	3.45 (± 0.37)	3.55* (± 0.38)	3.30 (± 0.58)	3.36 (± 0.32)	3.45 (± 0.37)	3.46 (± 0.40)
**leukocytes [Giga/L]**	3.63 (± 0.72)	3.53 (± 0.35)	3.71 (± 0.31)	3.63 (± 0.72)	3.53 (± 0.35)	3.71 (± 0.31)
**platelets [Giga/L]**	168.2 (± 24.79)	168.8 (± 20.83)	165.7 (± 19.38)	134.8 (± 39.81)	134.3 (± 58.1)	158.5 (± 26.31)
**hemoglobin [g/dL]**	10.0 (± 1.35)	10.2 (± 1.37)	9.5 (± 1.63)	9.81 (± 1.22)	10.05* (± 1.36)	10.07* (± 1.30)
**hematocrit [L/L]**	0.28 (± 0.02)	0.29 (± 0.02)	0.27 (± 0.04)	0.28 (± 0.02)	0.28 (± 0.03)	0.28 (± 0.03)

coagulation parameter	baseline			30 min		
**parameter**	**Jonosteril**	**Gelafundin**	**Tetraspan**	**Jonosteril**	**Gelafundin**	**Tetraspan**
**Quick [%]**	50.83 (± 6.04)	49.50 (± 6.68)	52.67 (± 6.47)	53.50 (± 8.09)	49.83 (± 8.65)	55.00 (± 7.40)
**aPTT [s]**	57.33 (± 1.83)	58.17 (± 5.49)	55.67 (± 6.71)	46.50 (± 5.50)	52.17* (± 5.56)	48.17 (± 4.75)
**INR**	1.51 (± 0.16)	1.55 (± 0.15)	1.50 (± 0.14)	1.46 (± 0.13)	1.56 (± 0.17)	1.41 (± 0.13)

Gelatin 4% and hydroxyethyl starch (130/0.42) impair clot formation in a human ex vivo whole-blood resuscitation model

Marco Mannes[1], Doreen Tabea Spiegelburg[1], Thomas Datzmann[1], Anke Schultze[1], Andreas Bauer[2], Gerhard Achatz[2], Markus Huber-Lang[1], Christian Karl Braun[3,4]

[1] Institute of Clinical and Experimental Trauma Immunology, Ulm University Medical Center, Ulm, Germany

[2] Department of Trauma Surgery and Orthopaedics, Reconstructive and Septic Surgery, Sportstraumatology, Trauma Surgery Research Group, German Armed Forces Hospital, Ulm, Germany

[3] Department of Pediatrics and Adolescent Medicine, Ulm University Medical Center, Ulm, Germany

[4] German Center for Child and Adolescent Health (DZKJ), Partner Site Ulm, Ulm, Germany

Supplemental Methods

Suppl. Methods 1 – Murine Model of Thorax Trauma and Hemorrhagic Shock
For the present study we retrospectively analyzed data only from the thorax trauma subgroups (TxT), that received volume resuscitation with either jonosteril or gelafundin. Male C57BL/6J mice (10–14 weeks, ~25 g) were housed in groups of five with food and water ad libitum under controlled temperature, humidity, and 14:10 h light–dark cycle. All procedures were approved by the local animal care committee and federal authorities (Reg.-No./Approval: 1409) and complied with the NIH Guide for the Care and Use of Laboratory Animals (2011).

Buprenorphine (0.05 mg/kg s.c.; Movianto, Germany) was given 30 min before trauma. Anesthesia was induced with 3% sevoflurane (Sevorane; Abbott, Germany) in oxygen. Blunt bilateral lung contusion was produced by a standardized blast wave to the chest, followed by continuous temperature control (37°C). The left femoral artery was cannulated for hemodynamic monitoring and pressure-controlled hemorrhage to a mean arterial pressure (MAP) of 30 ± 5 mmHg for 60 min, with reinfusion of heparinized shed blood over 15 min and subsequent infusion of either 2x 500 µL Jonosteril (Servoprax, Germany) or 2x 500 µl of Gelafundin (4%; Braun, Germany). For the present study, only mortality, heart rate and mean arterial pressure were analyzed for the respective groups.

We retrospectively compared the data from pilot experiments analyzing the resuscitation with Gelafundin with a control sub-group receiving Jonosteril, that was previously published [1].

Suppl. Methods 2 – ex vivo Resuscitation Model with crystalline or colloid solutions
We simulated volume resuscitation ex vivo with either crystalline solution (*Jonosteril*; Fresenius Kabi, Germany) or colloid solutions (*Gelafundin*; Gelafundin® ISO 40 mg/ml, Braun, Germany; or *Tetraspan*; hydroxyethyl starch 6%, Tetraspan 60 mg/ml, Braun, Germany) at various dilution ratios in human whole blood. See Suppl. Tab. 1 for constituents and chemical properties of the respective solutions.

For the ex vivo hWBM, blood was drawn from healthy probands (without current medication or known inflammation) into neutral monovettes Sarstedt, Germany) preloaded with fondaparinux (FPX; Arixtra®; Aspen Pharma Trading Ltd., Ireland) for a final FPX concentration of 8 µg/ml. Then respective volumes of whole blood were transferred to to 2-methacryloyloxethyl phosphorylcholine polymer (MPC, Lipidure®; NOF Corporation, Japan) coated 2 ml tubes preloaded with respective volumes of crystalline or colloid solution (or a combination of both, depending on the experimental set-up). For a ratio of 3:7 for example, 300 µl of volume solution per 1000 µl sample volume were added to 700 µl of FPX-anticoagulated whole blood per 1000 µl of sample volume.

Samples with respective experimental conditions were then either directly processed ("baseline" read-outs) or incubated simultaneously at 37°C on a spinning wheel under constant motion for up to 30 min (Suppl. Fig 2). After incubation, samples were either processed for blood gas analysis and/or pipetted into 3.2% citrated tubes (Sarstedt), Eppendorf tubes with 0.5 M Ethylenediaminetetra-acetic acid (EDTA; Sigma-Aldrich, Germany) or to HBSS–/– buffer containing tubes for further processing (see respective sections and Suppl. Fig 2).

Suppl. Methods 3 – ELISA analysis
After incubation, respective sample volumes were collected and immediately mixed with EDTA to a final concentration of 10 mM: Samples were then centrifuged at 800 g for 5 min at 4°C, the resulting supernatant was carefully collected and centrifuged again at 16.000 g for 2 min at 4°C. Plasma samples were frozen and stored at -80°C until further processed. For processing, samples were thawed and kept on ice throughout.

The following, commercially available ELISA kits were used, strictly adhering to the manufacturer's instructions: C3a ELISA kit (A031; Quidel, USA), C5a ELISA kit (EIA-3327; DRG international, USA), sC5b-9 ELISA kit (558315, BD Biosciences, USA), TAT ELISA (thrombin-antithrombin complexes; ab108907; Abcam, USA), human vWF-A2 DuoSet (von-Willebrand factor; DY27664-05, R&D systems, USA), sCD154 (=sCD40L; Human CD40 Ligand DuoSet; DY617, R&D systems, USA). Absorption was measured at 450 nm wavelength Tecan Sunrise Reader; Tecan Tradding AG, Switzerland).

Suppl. Methods 4 – Flowcytometry
After ex vivo incubation, respective sample volumes were diluted 1:12.5 in pre-warmed HBSS–/– buffer solution. Then, either PBS–/– (controls), ADP (Cat.-No.: A2754; Sigma Aldrich, Germany; final concentration: 5 µM), or TRAP-6 (Cat.-No.: T5045-02A.5, Biomol, Germany; final concentration: 32 µM) were added to 40 µl of each sample, carefully mixed and incubated for 10 min at 37°C. Subsequently, samples were stained for CD62P (final concentration: 2 µg/ml; Cat.-No.: 304904; BioLegend, USA) or for CD63 (final concentration: 1 µg/ml; Cat.-No.: 353004; BioLegend, USA) for 5 min at 37°C. After staining, samples were transferred to tubes containing 2 ml of HBSS–/– buffer solution, further diluted at 1:2 with HBSS–/– buffer solution and analyzed, using a FACSLyric 2 (BD, USA).

Gating Strategy
Platelets in whole blood were gated as described before [2]. In brief, singlets were identified via FSC-H/FSC-A plot and the platelet population was identified by size via FSC-A/SSC-A plot and analyzed for CD62P expression (mean fluorescence intensity, MFI).

[1] Halbgebauer R, Schult L, Borgel O, Maes A, Weißhaupt F, Rastner C, et al. Pathophysiological response in trauma-related acute kidney injury after blunt thoracic trauma and hemorrhagic shock in male mice. Am J Physiol-Ren Physiol. 2025;329: F311–F322. doi:10.1152/ajprenal.00029.2025
[2] Mannes M, Pechtl V, Hafner S, Dopler A, Eriksson O, Manivel VA, Wohlgemuth L, Messerer DAC, Schrezenmeier H, Ekdahl KN et al: Complement and platelets: prothrombotic cell activation requires membrane attack complex-induced release of danger signals. Blood Adv 2023, 7(20):6367-6380.
